# 

**Published:** 1858-10

**Authors:** 


### NOTICES, REVIEWS, AND VARIETIES.

*Farm, Garden, and Orchard Manuals*—sent, post-paid, for thirty cents each, by FOWLER & WELLS, 308 Broadway, New York—are among the most useful publications of the times; worthy of a place in every farm-house.

A CORRESPONDENT says: That yeast made of cider while it is fermenting, will make a light bread superior to any used.

TILDEN & Co.'s *Book of Formulæ*, of 162 pp., 8vo, 98 John street, New York—sent, post-paid, for one dollar—is a most valuable aid to druggists and country physicians, who have to prepare their own medicines. The preparations of TILDEN'S laboratory, at New Lebanon, N. Y., have a deserved reputation, among our best druggists, for purity and efficiency: hence, the prosperity which enables our liberal and courteous neighbor to keep his "coach and four" dappled grays—the admiration of all who see them. Seems to us, that making medicine is immensely more profitable than taking it, or prescribing it either.

S. H. MORRILL, a doctor and a poet, has written a book, in rhyme, on the means of health, containing more real, practical common-sense, than we supposed could be expressed in poetry. It is pretty hard work to make it jingle, sometimes; but, by hook or crook, it never fails to come.

M. L. KNAPP, M. D., President of Iowa University, has published three 8vo essays, 100 pp. each, on the unity of disease—that cholera, cholera infantum, and nurse's sore mouth, are a "lesion of nutrition," essentially scurvy, and should be treated accordingly. Learning, research, and medical acumen are evidenced in these volumes; and we hope the venerable President will succeed in drawing that attention to the subject which its importance demands.

*Peterson's Magazine*, for October, Philadelphia—two dollars a year—is ahead of all the monthlies, indicating industry, energy, and thrift. *Graham*, for October, comes as usual, promptly and without fail.

*Moore's Rural New Yorker*, Rochester, weekly—two dollars a year—has a wider circulation than any other agricultural publication in America. It merits public appreciation.

*American Agriculturist*, monthly, one dollar a year, New York, gives more valuable reading for a dollar than any of its cotemporaries; and, by its merit, has secured an unexampled success.

"*Western Water-Cure Journal*," Cleveland, Ohio, contains an article from Mrs. GROSS, "To Mothers," written with intelligence and unusual ability. It may be most profitably read by every young mother.

